# Meta‐analysis fails to show any correlation between protein abundance and ubiquitination changes

**DOI:** 10.1002/2211-5463.70197

**Published:** 2026-01-24

**Authors:** Nerea Osinalde, Unai Alduntzin, Gorka Prieto, Ugo Mayor

**Affiliations:** ^1^ Department of Biochemistry and Molecular Biology Faculty of Pharmacy, UPV/EHU Vitoria‐Gasteiz Spain; ^2^ Department of Biochemistry and Molecular Biology Faculty of Science and Technology, University of the Basque Country (UPV/EHU) Leioa Spain; ^3^ Department of Communications Engineering University of the Basque Country (UPV/EHU) Bilbao Spain; ^4^ Ikerbasque, Basque Foundation for Science Bilbao Spain

**Keywords:** correlation, mass spectrometry, proteome, ubiquitome

## Abstract

Ubiquitination serves as a key regulatory mechanism in nearly all cellular processes, though it was originally identified as a post‐translational modification that targets proteins for degradation. Accordingly, these two molecular events have since been tightly linked, and changes in protein abundance are frequently interpreted as indirect indicators of ubiquitination dynamics. Nevertheless, the relationship between protein abundance and ubiquitination has not been systematically examined across distinct biological systems. Here, we conducted a comprehensive meta‐analysis to assess the correlation between protein abundance and ubiquitination levels determined by mass spectrometry in mammals, plants, and yeast. Quantitative proteomics and diGly‐ubiquitin peptides data from 19 independent studies encompassing over 50 experimental conditions were analyzed. The findings indicate that alterations in protein abundance cannot reliably be used to infer ubiquitination events – and vice versa – highlighting the need for caution when interpreting proteomic data as a proxy for ubiquitin‐mediated regulation.

## Graphical abstract

We analyzed over 50 published proteomics datasets to explore the relationship between protein levels and ubiquitination changes across multiple experimental conditions and biological systems. Although ubiquitination is often associated with protein degradation, our analysis shows that changes in ubiquitination do not globally correlate with changes in protein abundance and *vice versa.*

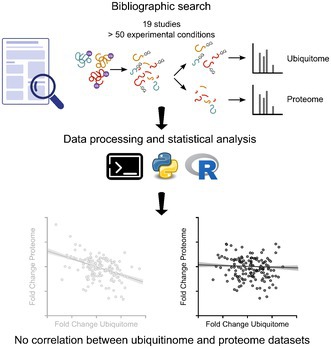

**Abbreviations**
DDA: data‐dependent acquisition
DIA: data‐independent acquisition
TMT: tandem mass tag
UPS: ubiquitin proteasome system

Cellular protein abundance reflects the dynamic equilibrium among multiple interconnected processes, ranging from gene transcription and mRNA processing to translation, post‐translational modification, and protein degradation. Historically, mRNA levels were assumed to be the primary determinant of protein abundance, and transcriptomic data were therefore used to infer protein‐level changes. Nevertheless, advances in next‐generation sequencing and mass spectrometry have demonstrated that mRNA abundance only partially predicts protein levels [1, 2].

Analogously, protein degradation has traditionally been associated with protein ubiquitination. Indeed, ubiquitination was first identified as a signal for proteasomal degradation [3], and the Nobel Prize in Chemistry in 2004, awarded to Aaron Ciechanover, Avram Hershko, and Irwin Rose, further emphasized the fundamental link between protein ubiquitination and degradation [4].

Ubiquitin is a small, 76‐amino acid protein that, through the coordinated action of E1 activating, E2 conjugating, and E3 ligase enzymes, can be covalently attached to target proteins or other ubiquitin molecules already linked to those proteins. Polyubiquitin chains form when the C‐terminus of one ubiquitin moiety conjugates to a lysine residue (K6, K11, K27, K29, K33, K48, and K63) or the N‐terminal methionine (Met1) on another ubiquitin. Depending on the linkage pattern, ubiquitin chains can adopt homogeneous, heterogeneous, or branched topologies [5]. The functional outcome of ubiquitination depends not only on the modified substrate residues but also on the chain architecture [6]. For instance, K63‐linked chains participate in DNA repair, endocytosis, and immune signaling [7, 8, 9]; while K11‐, K27‐, and K29‐linked chains are involved in cell division, DNA damage, and proteotoxic stress response, respectively [10, 11, 12]. Among these, K48‐linked chains are the most extensively characterized, serving as the canonical signal for proteasomal degradation [13].

The ubiquitin proteasome system (UPS) is the primary pathway for intracellular protein turnover, responsible for degrading approximately 80–90% of cytosolic proteins in mammalian cells [14]. The UPS primarily targets short‐lived regulatory proteins, including cell cycle regulators, transcription factors, and damaged or misfolded proteins. In turn, autophagy degrades long‐lived proteins, insoluble aggregates, and organelles [15, 16, 17, 18]. Together, the UPS and autophagy work in concert to maintain cellular proteostasis [19].

Despite extensive research on ubiquitination and protein turnover, a systemic evaluation of the correlation between ubiquitination levels and protein abundance across diverse biological systems has not been performed. Here, we address this gap by performing a meta‐analysis of quantitative proteomic and ubiquitomic data from 19 large‐scale studies, encompassing over 50 experimental conditions (Table 1). Our analyses reveal a striking lack of correlation between changes in protein abundance and ubiquitination levels, indicating that alterations in protein levels determined by proteomics cannot be reliably predict alterations in ubiquitination, challenging a long‐standing assumption in molecular biology.

**Table 1:** Summary of the research papers in which differential proteomics and ubiquitomics studies were carried out, and consequently, were selected for the present study.

Cluster	Author/year	Biological study	Q‐MS method	*p*‐values available	Time course	Ref
Plants	Guo *et al*. (2017)	Ethylene‐induced senescence	Label free	✓	–	[20]
	He *et al*. (2019)	Rice seed germination	Label free	✓	–	[21]
	Liu *et al*. (2022)	Influence of Tª on flowering	TMT	✓	–	[22]
	Lu *et al*. (2019)	Flowering stage 3vs5	Label free	–	–	[23]
Animals	Abreha *et al*. (2019)	Alzheimer	Label free	✓	–	[24]
	Baher *et al*. (2019)	MuRF1 overexpression	Label free	✓	–	[25]
	Dybas *et al*. (2019)	Stimulation of T cells	SuperSILAC	–	–	[26]
Fungi	Iesmantavicious *et al*. (2014)	Rapamycin treatment	SILAC	–	–	[27]
UPS alterations	Akimov *et al*. (2018)	Bortezomib and AP15 treatment	Label free	–	–	[28]
	Kim *et al*. (2011)	Bortezomib treatment	SILAC	–	✓	[29]
Cell lines	Agarwall *et al*. (2021)	Parkin	SILAC	✓	–	[30]
	Rusilowicz‐Jones *et al*. (2020)	UP30	SILAC	–	–	[31]
	Hansen *et al*. (2021)	Released from synchronization	DIA	–	✓	[32]
	Li *et al*. (2018)	Gefitinib resistance	SILAC	–	–	[33]
	Ordureau *et al*. (2020)	Antimycin/Oligomycin treatment	TMT	✓	✓	[34]
	Rose *et al*. (2016)	Parkin and variants	TMT	–	–	[35]
	Steger *et al*. (2021)	FT671 treatment	DIA	–	✓	[36]
	Theurillat *et al*. (2014)	SPOP E3 ligase	SILAC	–	–	[37]
	Wu *et al*. (2015)	SAHA treatment	SILAC	–	–	[38]

## Material and methods

### Bibliographic search

A comprehensive search of the PubMed® database was conducted in 2023 to identify original research articles that included both quantitative proteomics and ubiquitomics analyses under identical experimental conditions. Only studies providing accessible quantitative data – either deposited in public repositories or presented in supplementary materials – were considered for further analysis. To ensure analytical consistency, papers were included only if they reported complete protein‐level and diGly site quantitative data suitable for statistical evaluation (File S1).

### Data processing and statistical analysis

Data processing and statistical analysis were performed using either R (version 4.3.0) with *tidyverse* package (version 2.0.0) or using Python (version 3.7.9) with the *scipy* package (version 1.7.3). Quantitative data extracted from supplementary tables of selected studies were consolidated into a unified dataset (File S2) comprising six columns: (i) study identifier and experimental condition, (ii) protein identifier, (iii) protein‐level fold change between conditions, (iv) corresponding *p*‐value, (v) fold change of each ubiquitination site (*individual diGly‐containing peptide*), and (vi) associated *p*‐value. Studies lacking *p*‐values when required were excluded. When overall fold change values were not reported, they were computed by averaging replicate intensities. Proteins were retained for analysis only if intensity data were available for more than half of the total biological replicates (rounded up). In cases where a protein was detected in all replicates of one condition but absent in the other, the minimum detected intensity in the condition with missing values was used to calculate the ratio.

From this initial dataset, a secondary table (File S3) was generated by averaging the log_2_ ratios of the distinct diGly‐modified peptides reported for the same protein within the study (*average diGly‐containing peptide ratio*).

Two‐sided Pearson correlation coefficients and associated *p*‐values were calculated using the *cor.test* function of the R *stats* package with default parameters. Correlations were computed under four different conditions for each study (Table S1): (a) using the averaged ubiquitination values of all identified proteins. (b) Considering only up‐ubiquitinated proteins. (c) Considering only down‐ubiquitinated proteins. (d) Using all individual (nonaveraged) ubiquitination site values. Unless otherwise specified, threshold values of ± log_2_ (1.5) for fold changes and 0.05 for *p*‐values were applied. Hierarchical clustering was performed using group‐average method on Euclidean distances implemented in *scipy*. Only proteins displaying fold change values exceeding ± log_2_ (1.5) in the proteome were included in the clustering analysis.

The completed PRISMA checklist is provided as Supporting Information (File S4).

## Results

To systematically evaluate whether protein ubiquitination directly influences protein abundance across biological systems, we analyzed 19 independent proteomic datasets that included simultaneous quantitative measurements of both the proteome and ubiquitome under identical experimental conditions. Quantitative data were extracted from each study and subjected to correlation analysis across all proteins for which both protein‐level and ubiquitination‐level data were available. Specifically, we examined the relationship between changes in protein abundance and corresponding diGly peptide ratios.

We systematically searched for original research articles in which investigators performed both differential proteomics and ubiquitomics analyses under the same experimental conditions. Studies were included only if the complete mass spectrometry (MS) datasets, including quantitative information, were uploaded in public repositories or provided as supplementary materials. A total of 19 publications met these inclusion criteria.

The selected studies encompassed a wide range of biological systems, and we grouped them according to the organism in which the experiments were performed to account for potential species‐specific differences in proteostasis. Accordingly, the 19 studies were classified into four categories: plants (*n* = 4), animals (*n* = 3), yeast (*n* = 1), and human cell lines (*n* = 11). Within the human cell line category, we included an additional subgroup comprising two studies in which the UPS was pharmacologically inhibited, as these interventions could directly influence ubiquitination–protein‐level relationships.

In addition to differences in biological systems, the studies also varied in the mass spectrometry (MS)‐based approaches employed. Most studies used data‐dependent acquisition (DDA), in which the most abundant peptides in each scan are selected for fragmentation, allowing identification and quantification. Two studies – one on circadian biology in HEK293 and U2OS cells [32] and another characterizing USP7 deubiquitinase substrates [36] – used data‐independent acquisition (DIA), a method in which all peptides within a defined mass range are fragmented simultaneously, improving reproducibility and coverage.

Regarding labelling strategies, most human cell line [29, 30, 31, 33, 37, 38] and yeast [27] studies relied on stable isotope labelling with amino acids in cell culture (SILAC) to quantify changes in both proteome and ubiquitome abundance. Isobaric labelling via tandem mass tag (TMT) was employed in a study investigating PARKIN‐ and PINK1‐dependent mitophagy in HTC116 cells [35], as in another analyzing PARKIN‐mediated ubiquitination in induced neurons [34]. Among all cell line studies, only one applied a label‐free mass spectrometric approach [28]. Notably, this study did not utilize the conventional anti‐diGly antibody, which specifically recognizes the diglycine remnant left on ubiquitinated peptides following trypsin digestion. Instead, the authors developed UbiSite antibody, which targets the C‐terminal 13 amino acids of ubiquitin that remain conjugated to peptides after endoproteinase LysC digestion.

Regarding the studies performed in animals and plants, most relied on label‐free quantification to monitor changes in protein and ubiquitination abundance [21, 23, 24, 25]. A study on the proteome of tree peony (*Paeonia suffruticosa*) cut flowers subjected to different temperatures used TMT labelling to investigate the effect of temperature on senescence and abscission [22]. Similarly, in a study examining the role of ethylene – a plant hormone involved in senescence – on the ubiquitome and proteome of petunia corollas, TMT labelling and label‐free quantitative strategy were used, respectively [20]. The superSILAC approach was employed to assess proteome and ubiquitome changes in activated CD4^+^ T cells [26].

Although the selected studies employed distinct MS‐based quantitative strategies, all were processed using a unified analytical workflow. When quantitative data of biological replicas were available, proteins were retained only if detected in more than half of the replicates, allowing calculation of an average fold change (File S2). Similar criteria were applied to diGly peptide datasets (File S3). Finally, we computed Pearson correlation coefficients for each of the 57 experimental conditions represented across the 19 studies. For three additional experiments – corresponding to studies that reported only deregulated proteins – we also calculated Pearson correlation coefficients and *p*‐values (Table S1).

### Poor correlation between protein abundance and corresponding diGly‐containing peptide ratios

**Fig. 1:**
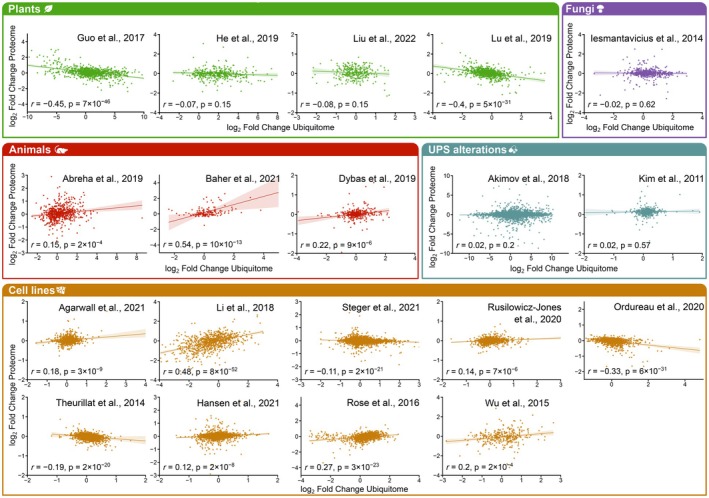
Proteomics quantified protein abundance changes do not inversely correlate with changes in diGly peptide abundance. Data from 19 independent proteomic experiments on which both protein and ubiquitome abundance levels were analyzed and quantified under two different conditions are shown. Datasets have been grouped in five major clusters: Investigations carried out in plants are colored in green, whereas investigations done in animals, yeast, and cell lines are marked in red, purple, and yellow, respectively. Additionally, two studies performed in cell lines but in which UPS was altered are colored in blue. Each point corresponds to one protein from the dataset, as retrieved from the corresponding papers and data repositories. Ubiquitome data are shown as an average of all diGly peptides quantified for each protein. For each dataset, two‐sided Pearson correlation coefficients and associated *p*‐values were calculated. In the case of articles with more than one experimental condition, only the one with the correlation closest to the expected trend is shown.

Figure 1 illustrates the correlation between proteome and ubiquitome changes across the 19 research articles analyzed, highlighting the strongest when multiple experimental conditions were tested within a single study. Additional comparisons are provided in Fig. S1.

If protein ubiquitination directly triggered proteasomal degradation, a negative correlation would be expected – that is, reduced protein abundance accompanied by increased ubiquitination. Conversely, under conditions where the UPS is inhibited, a positive correlation might occur due to the simultaneous accumulation of proteins and their ubiquitinated versions. Across all biological systems examined and regardless of the MS‐based quantification method used, no consistent correlation was observed between global proteome and ubiquitome changes. Only two plant studies investigating senescence [20, 23] exhibited a weak negative correlation. Specifically, ethylene‐treated *Petunia hybrid* petals, in which ethylene promotes senescence [20], displayed increased ubiquitination concomitant with reduced protein abundance. Similarly, in *Rosa hybrid*, protein levels were modestly inversely correlated with ubiquitination during late stages of flower opening, coinciding with the onset of senescence [23]. Nonetheless, in both cases, the negative correlations were relatively weak (Pearson *r* = −0.45 and *r* = −0.4, respectively).

### Correlation does not improve when considering diGly‐containing peptide ratios individually

A single protein can be ubiquitinated at multiple lysine residues, and the modification dynamics of each site may differ substantially [28, 34]. Therefore, averaging the ratios of all ubiquitinated peptides corresponding to the same protein could mask the contribution of specific regulatory ubiquitination sites. To explore this possibility, we compared the fold change ratio of each protein with that of every individual diGly‐modified peptide detected for that same protein and recalculated the Pearson correlation coefficient (Table S1).

**Fig. 2:**
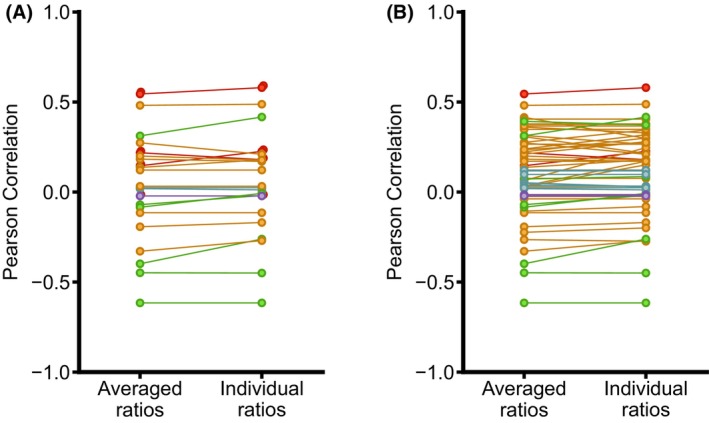
Changes in protein and ubiquitination abundance do not correlate better considering individual diGly site ratios. Given the possibility that independent diGly peptide behavior would better conform to the predicted correlation with overall protein abundance, data were reanalyzed for all the datasets compiled in this work. Pearson correlation was calculated between changes in protein abundance and each diGly peptide corresponding to the protein for all the experimental conditions analyzed. Changes in Pearson correlation when considering average or individual diGly ratios are shown for (A) the articles and conditions shown in Fig. 1 and (B) the rest of the experimental conditions. No improvement was observed with this further analysis, further confirming that changes in protein abundance cannot be used as a predictor of protein ubiquitination, nor globally, neither for specific ubiquitination sites. *n.s., not statistically significant.

In a few instances, the correlation became slightly more negative when individual diGly‐modified peptide ratios were analyzed (Fig. 2). However, the differences were minor, with Pearson's *r* values changing by less than 5%. These findings indicate that the overall correlation between global proteome and ubiquitome abundance remains largely unchanged regardless of whether averaged or individual ubiquitinated peptide ratios are used in the calculations. Therefore, in subsequent analyses, average ubiquitinated peptide ratios were used.

### Lack of correlation between significantly regulated proteins and corresponding ubiquitination sites

The quality of the quantitative data can influence the observed relationship between protein abundance and corresponding diGly peptide ratios. To minimize noise from statistically insignificant changes, we recalculated Pearson correlation coefficients using only proteins with significant abundance changes (*p* < 0.05).

**Fig. 3:**
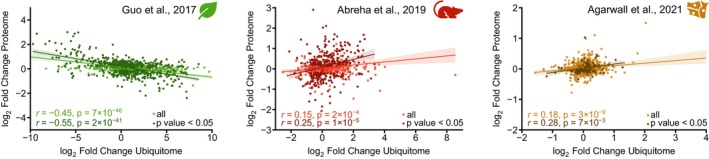
Statistically significantly regulated proteins neither correlate with changes in ubiquitination. Pearson correlation was calculated between ratios of proteins displaying a *p*‐value < 0.05 and the average ratio of their corresponding diGly sites. The most representative study for each cluster reporting a *p*‐value in the proteomics data is shown. For each comparison, Pearson correlation and *p*‐value are calculated; confidence interval is shown as shaded area.

In the study by Guo *et al*. [20], the previously observed negative correlation (Fig. 1) became slightly stronger when only significantly regulated proteins were included (Fig. 3). However, in the remaining investigations, the correlation either remained largely unchanged or shifted in the opposite direction from the expected if ubiquitination were consistently associated with protein degradation (Fig. S2).

These results indicate that applying a significance threshold (*p* < 0.05) does not substantially affect the overall relationship between global protein and ubiquitination changes. Nevertheless, we propose that when protein degradation plays a central role in the biological process under investigation – such as in plant senescence – the inclusion of statistical significance as a filtering criteria may provide additional biological insight.

### Upregulated and downregulated proteins can be either more or less ubiquitinated

**Fig. 4:**
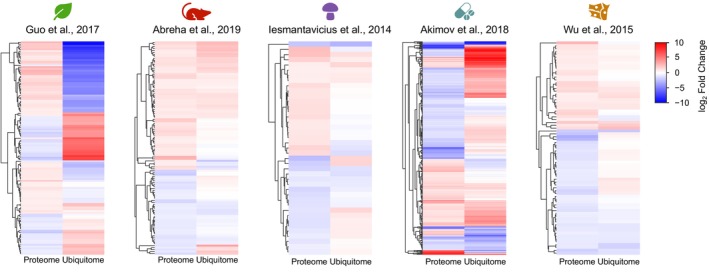
Downregulated and upregulated proteins are not more and less ubiquitinated, respectively. Hierarchical clustering heatmap of proteome and ubiquitome of proteins displaying a fold change above 1.5 or below 0.67. Each row represents a protein from the study. The color code indicates the protein abundance and ubiquitination levels in log_2_ scale. A representative study for each experimental system is shown. Only for the work of Guo and colleagues, distinct groups of downregulated and more ubiquitinated and upregulated and less ubiquitinated can be found.

K48‐linked polyubiquitinated chains typically serve as a signal for proteasomal degradation [39], and consequently, decreased protein levels are often interpreted as a consequence of ubiquitin‐dependent degradation. In this context, all downregulated proteins would be expected to exhibit increased ubiquitination, whereas upregulated proteins should display low levels of ubiquitination. To test this hypothesis, we evaluated the predicted relationship between protein abundance and ubiquitination by hierarchical clustering, color‐coding upregulated and downregulated protein fold changes (FC ≥ 1.5 and FC ≤ 0.67, respectively) and corresponding average diGly ratios for visual assessment. Consistent with previous observations (Figs 1 and 3), the two studies on plant senescence [20, 23] showed a weak trend suggesting that ubiquitination may influence protein abundance to some extent (Fig. 4). However, across the remaining studies with available data, no consistent relationship was observed between changes in ubiquitination and corresponding changes in protein levels (Fig. 4 and Fig. S3).

**Fig. 5:**
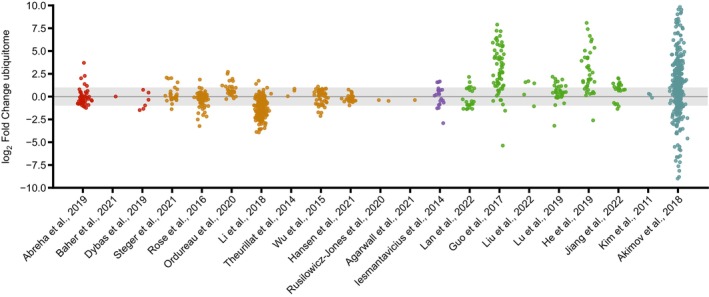
Downregulated proteins contain either up‐, down‐, or nonregulated diGly peptides. For each paper analyzed, proteins displaying a fold change below 0.67 were selected and the ratio of each of their corresponding diGly peptide was plotted.

We further assessed this hypothesis by focusing specifically on the ubiquitination status of downregulated proteins (log_2_ fold change < −0.66). For this analysis, three additional studies were included, as they provided data only for deregulated proteins. Two of these investigations were conducted in plants and examined rose petal abscission [40] and metabolic leaf senescence [41], while the third, performed in human cell lines, analyzed proteins regulated by the deubiquitinase UPS14 [42]. As shown in Fig. 5, with the exception for the two studies on plant senescence [20, 23] and one on rice seed germination [21], downregulated proteins were not more ubiquitinated in any of the remaining 19 studies analyzed.

### Time delays do not explain the lack of correlation between protein and ubiquitination levels

Having ruled out a direct correlation between protein abundance and ubiquitination levels, we next hypothesized that temporal delays might underlay their apparent lack of association, similar to the well‐documented time lag between mRNA and protein expression changes [43]. To evaluate this possibility, we assessed whether nonsynchronous correlations – reflecting potential delays in proteasome‐dependent protein degradation – could explain the observed patterns. Specifically, we compared global protein and ubiquitination levels across different time points in studies that included time course experiments.

**Fig. 6:**
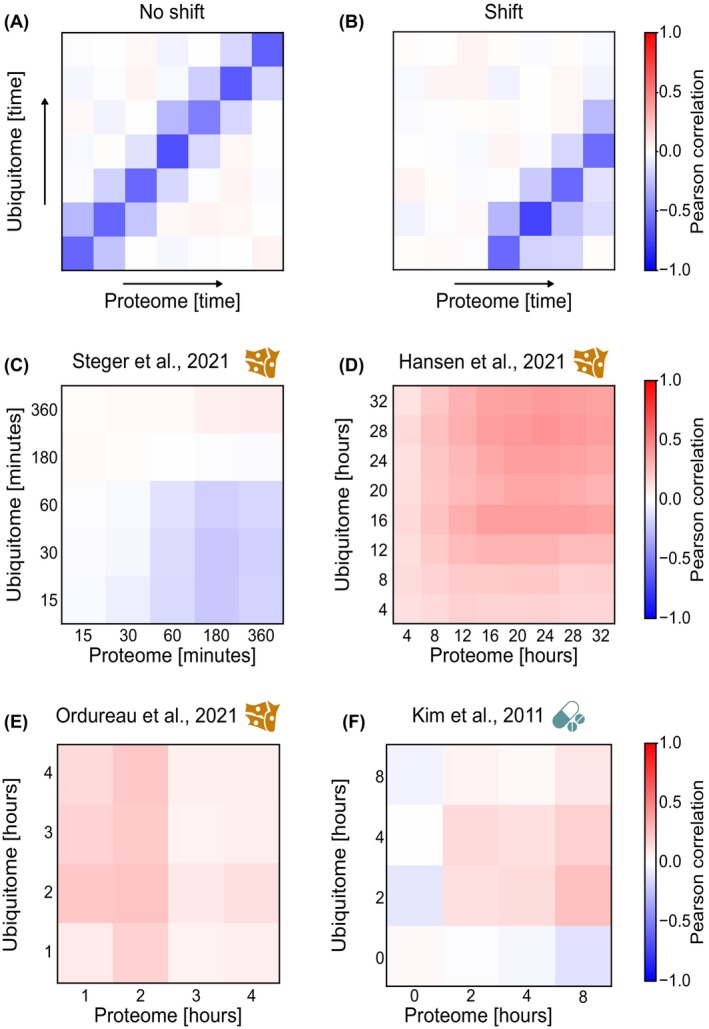
Heatmap of Pearson correlation coefficient between protein abundance and ubiquitination levels of time course experiments. Theoretical representation of negative correlation between protein and ubiquitin changes with (A) no time shift and (B) with shift. (C–F) Correlation of the time course experiments in which proteomics and ubiquitome data were available.

Among the datasets analyzed, four studies incorporated temporal profiling of both the proteome and ubiquitome. To conceptualize the expected behavior, we first illustrated a theoretical model using heat maps depicting two possible scenarios: (a) a negative correlation between protein and ubiquitination levels at the same time point (Fig. 6A), and (b) a delayed negative correlation, in which changes in ubiquitination precede changes in protein abundance (Fig. 6B).

Applying this approach to the four available time course datasets revealed only weak evidence of such temporal relationships. A mild pattern consistent with a potential time lag was observed in cells treated with a USP7 deubiquitinase (DUB) inhibitor [36] (Fig. 6C). In contrast, no comparable trends were detected in cells exposed to a USP30 DUB inhibitor [34] (Fig. 6E), nor in those subjected to proteasome inhibition with bortezomib [29] (Fig. 6F). Likewise, proteome and ubiquitome oscillations measured across the circadian cycle showed no time‐dependent association [32] (Fig. 6D).

## Discussion

Since the discovery by Chau *et al*. [44] in 1989 that K48‐linked polyubiquitin chains target substrates for proteasomal degradation, ubiquitination has been closely associated with protein turnover. This association has become so entrenched that changes in ubiquitination are often interpreted as indicative of protein degradation, even in the absence of direct experimental evidence. However, accumulating evidence over the past decades has revealed that ubiquitin serves regulatory roles far beyond proteasomal targeting, including protein–protein interactions, protein localization, and endocytosis [45, 46, 47]. In agreement with this expanded view, our meta‐analysis demonstrates that, except under very specific biological conditions, there is no consistent association between changes in proteome and ubiquitome levels.

A key methodological consideration is that most ubiquitome studies included in this meta‐analysis is that most of them use the K‐*ε*‐GG enrichment to capture ubiquitinated peptides. This approach enriches all ubiquitinated peptides regardless of chain type. Consequently, this method does not distinguish between different forms of ubiquitination, such as K48‐linked polyubiquitination – which primarily targets proteins for proteasomal degradation – and K63‐linked polyubiquitination, which is typically associated with nondegradative processes including signal transduction, DNA repair, and endocytic trafficking [7, 8, 9]. Monoubiquitinated peptides are also enriched alongside polyubiquitinated species, preventing assessment of the relative abundance of specific chain linkages. Therefore, while our analysis provides a comprehensive and site‐specific overview of global ubiquitination dynamics, results should be interpreted as reflecting general ubiquitination changes rather than specific chain architectures. Future studies using linkage‐specific antibodies or targeted MS strategies will be valuable to determine how particular ubiquitin chain architectures contribute to the observed regulatory effects.

Proteins typically contain multiple lysine residues, providing the potential for several ubiquitination sites. However, not all lysines are equally susceptible to modification, as E3 ubiquitin ligases recognize motifs that depend not only on the primary sequence surrounding the target lysine but also on the protein's secondary and tertiary structure. Bioinformatics analyses indicate that ubiquitination sites tend to be surface‐exposed and are frequently located within loop regions of the proteins [48]. Despite substantial advances in identifying ubiquitination motifs [49, 50], only a few have been well‐characterized since the first linear ubiquitination motif was described in 1986 [51, 52]. Both large‐scale ubiquitome studies [28, 34] and single‐protein analyses [53] commonly identify multiple ubiquitination sites within a single protein, and the abundance of each site can vary depending on the experimental conditions. For instance, in a previous study, we identified six ubiquitination sites in DDI1, three of which (K77, K133, and K151) were less ubiquitinated in the absence of the E3 ligase UBE3A, whereas the remaining sites were unaffected, indicating site‐specific regulation by this enzyme [53].

Importantly, all the studies included in this meta‐analysis relied on bottom‐up MS, currently the gold standard for large‐scale protein analysis. In bottom‐up approaches, proteins are digested into peptides prior to MS analysis [54]. Consequently, protein isoforms cannot be distinguished and the information of multiple modified sites mapping to the same protein does not imply that these modifications co‐exist on a single proteoform. Top‐down MS, which analyzes intact proteins [55], would be necessary to resolve combinatorial ubiquitination patterns and clarify which proteins are truly targeted for degradation. In the absence of intact protein data, we assessed individual diGly peptide ratios to explore the relationship between protein abundance and site‐specific ubiquitination. To ensure our conclusions were not dependent on individual peptide ratios, we also assessed correlations using average diGly peptide ratios. Integrating data from 19 large‐scale studies covering over 50 experimental conditions, we consistently observed that changes in the proteome and ubiquitome show little to no significant correlation.

The investigations examined here span highly diverse biological systems – including human cell lines, animal models, yeast, and plants – and address topics such as Alzheimer's disease [24], muscle atrophy [25] cancer [30, 37], therapeutic responses [36, 38], *T*‐cell receptor stimulation [26], nutrient starvation [27], mitophagy [31, 34, 35] and proteasome inhibition [28, 29]. In several of these, the original authors already reported a lack of correlation between proteome and ubiquitome alterations [26, 28, 29]. Dybas *et al*. [26], for instance, reported that changes in TCR‐induced ubiquitination were largely independent of protein degradation, and pattern similarly observed in *Drosophila* [56]. Our comprehensive re‐analysis confirms that this absence of correlation is a widespread phenomenon, independent of the organism or biological process under study, suggesting it represents a general trend within the available data.

Two plant senescence studies represent a notable exception to this trend. Lu *et al*. [23] and Guo *et al*. [20] both report modest but consistent negative correlations between protein and ubiquitination levels (*r* = −0.40 and −0.45). These results imply that ubiquitin‐mediated regulation of protein homeostasis may play a more predominant role under specific physiological contexts, such as senescence. In most other conditions, however, ubiquitination serves regulatory or signaling functions independent of protein turnover. Consequently, protein abundance is likely regulated by additional mechanisms, preventing any correlation between ubiquitination and total protein levels.

Senescence is the final stage of plant organ development, characterized by the systematic breakdown of cellular components to recycle nutrients. Protein degradation is a central feature of this process, as proteins represent a major reservoir of nitrogen and other essential resources [17, 57, 58]. The ubiquitin proteasome system (UPS) and autophagy are the primary pathways mediating protein turnover during senescence, selectively removing damaged, misfolded, or regulatory proteins. It is well‐documented that the proteasome exhibits high activity in senescing leaves of oilseed rape and wheat [59, 60]. In *Arabidopsis*, disruption of proteasome subunits delays senescence, underscoring the essential role of proteasome‐dependent degradation [61]. Recent work by Lu *et al*. [62] has revealed that ethylene promotes petal senescence in roses by inducing the E3 ligase RhSAF, which ubiquitinates and degrades the GA receptor RhGID1. Together, these observations, supported by our meta‐analysis, reinforce that ubiquitin‐dependent protein degradation is a central mechanism driving plant senescence.

Nevertheless, the contribution of ubiquitin‐mediated protein degradation varies across species and the specific senescence conditions. For example, in tree peony, heat‐induced senescence showed no concordance between proteome and ubiquitome changes [22], and in *Arabidopsis thaliana*, depletion of the senescence‐related E3 ligase UPL3 did not result in correlated changes in protein abundance and ubiquitination [41]. Additionally, the moderate correlation observed in the studies by Guo *et al*. and Lu *et al*. suggests that, beyond the UPS, other degradation pathways – most likely autophagy – also play a significant role in protein turnover during petal senescence. Collectively, all these data indicate that protein degradation during plant senescence is orchestrated by multiple, partially independent pathways, with ubiquitin‐dependent mechanisms playing a central but context‐dependent role alongside other processes such as autophagy.

To further explore the relationship between ubiquitination and protein abundance, our analysis included two studies in which the ubiquitin–proteasome system (UPS) was pharmacologically inhibited [28, 29]. In these datasets, cells were treated with proteasome inhibitors (bortezomib and AP15) to block proteasomal degradation. If ubiquitination primarily reflected degradative targeting, one would expect a positive correlation between increased ubiquitination and protein accumulation under these conditions. However, we did not detect any consistent correlation between ubiquitination and protein abundance in either study. Notably, the SILAC‐based time course data from Kim *et al*. [29] also showed no correlation even when potential temporal delays were considered. These findings reinforce that global ubiquitination levels are not necessarily indicative of proteasomal degradation and support the broader view that ubiquitination serves diverse regulatory roles beyond protein turnover.

The temporal relationship between interconnected cellular events represents a critical consideration when interpreting molecular data. Sequential processes, such as gene transcription and protein translation, or protein ubiquitination and ubiquitin‐dependent protein degradation, may occur with distinct kinetics. Consequently, correlation between mRNA and protein levels, or between ubiquitination and protein abundance, may only be observable at specific time points. For example, during *Drosophila* embryogenesis, maximal mRNA‐protein correlation was detected between mRNA at 12 h and protein levels at 16 h [63]. Consistently, in our analysis of USP7 deubiquitinase inhibition, alterations in ubiquitination at 30 min exhibited a modest inverse correlation with protein‐level changes at 3 h, whereas no correlation was observed when both events were compared at the same time point. Notably, in the other three time course experiments analyzed, no temporal effects were detected. Taken together, these findings suggest that the general lack of concordance between changes in protein abundance and corresponding diGyl‐modified peptides is not attributable to time lag effects.

In summary, our meta‐analysis demonstrates that ubiquitination and protein abundance show little concordance, emphasizing that the presence of ubiquitination does not necessarily imply protein degradation, nor does protein abundance reliably reflect ubiquitination status. Thus, quantification of total protein levels by proteomics or other techniques, such as western blotting, is insufficient on its own to infer or suggest changes in protein ubiquitination status.

## Conflict of interest

The authors declare no conflict of interest.

## Author contributions

NO, UA, and UM: conceptualization; UA: visualization; GP: data curation and formal analysis; NO: writing‐original draft preparation; UM, UA, and GP: writing‐review and editing. All authors contributed to the article and approved the submitted version.

## Supporting information


**File S1.** PRISMA flow diagram.

**File S2.** Consolidated data obtained from each of the 54 experimental conditions included in the 19 analyzed research papers. Six columns are provided: (i) the name of the paper and the experimental conditions under study, (ii) the protein identifier, (iii) the protein‐level fold change between the experimental conditions, (iv) the *p*‐value associated with this fold change, (v) the ubiquitination‐level fold change between the experimental conditions, (vi) and the *p*‐value associated with this fold change.

**File S3.** Consolidated data after averaging the ubiquitination fold changes in logarithmic scale when different values were provided for the same protein in the same study. Four columns are provided: (i) the name of the paper and the experimental conditions under study, (ii) the protein identifier, (iii) the protein‐level fold change between the experimental conditions, and (iv) the ubiquitination‐level fold change between the experimental conditions.

**File S4.** PRISMA 2020 checklist.

**Table S1.** Two‐sided Pearson correlation coefficients and *p*‐values.

**Fig. S1.** Protein – diGly correlation analysis for experimental conditions other than the highest‐correlation condition.

**Fig. S2.** Pearson correlation between proteins and diGly peptides using significantly regulated proteins.

**Fig. S3.** Relationship between changes in down‐ and up‐regulated proteins and their corresponding diGly ratios.

## Data Availability

The data that support the findings of this study are available in the supplementary material of this article.

## References

[1] Vogel C and Marcotte EM (2012) Insights into the regulation of protein abundance from proteomic and transcriptomic analyses. Nat Rev Genet 13, 227–232. 22411467 10.1038/nrg3185 PMC3654667

[2] Abreu R d S , Penalva LO , Marcotte EM and Vogel C (2009) Global signatures of protein and mRNA expression levels. Mol BioSyst 5, 1512–1526. 20023718 10.1039/b908315d PMC4089977

[3] Varshavsky A (2006) The early history of the ubiquitin field. Protein Sci 15, 647–654. 16501229 10.1110/ps.052012306 PMC2249785

[4] Kresge N , Simoni RD and Hill RL (2006) The discovery of ubiquitin‐mediated proteolysis by Aaron Ciechanover, Avram Hershko, and Irwin Rose. J Biol Chem 281, e32–e36.

[5] Komander D and Rape M (2012) The ubiquitin code. Annu Rev Biochem 81, 203–229. 22524316 10.1146/annurev-biochem-060310-170328

[6] Akutsu M , Dikic I and Bremm A (2016) Ubiquitin chain diversity at a glance. J Cell Sci 129, 875–880. 26906419 10.1242/jcs.183954

[7] Liu P , Gan W , Su S , Hauenstein AV , Fu T , Brasher B , Schwerdtfeger C , Liang AC , Xu M and Wei W (2018) K63‐linked polyubiquitin chains bind to DNA to facilitate DNA damage repair. Sci Signal 11, eaar8133. 29871913 10.1126/scisignal.aar8133 PMC6434707

[8] Saeed B , Deligne F , Brillada C , Dünser K , Ditengou FA , Turek I , Allahham A , Grujic N , Dagdas Y , Ott T *et al*. (2023) K63‐linked ubiquitin chains are a global signal for endocytosis and contribute to selective autophagy in plants. Curr Biol 33, 1337–1345.e5. 36863341 10.1016/j.cub.2023.02.024

[9] Madiraju C , Novack JP , Reed JC and Matsuzawa S (2022) K63 ubiquitination in immune signaling. Trends Immunol 43, 148–162. 35033428 10.1016/j.it.2021.12.005 PMC8755460

[10] Wickliffe KE , Williamson A , Meyer H‐J , Kelly A and Rape M (2011) K11‐linked ubiquitin chains as novel regulators of cell division. Trends Cell Biol 21, 656–663. 21978762 10.1016/j.tcb.2011.08.008 PMC3205209

[11] Shearer RF , Typas D , Coscia F , Schovsbo S , Kruse T , Mund A and Mailand N (2022) K27‐linked ubiquitylation promotes p97 substrate processing and is essential for cell proliferation. EMBO J 41, e110145. 35349166 10.15252/embj.2021110145 PMC9058539

[12] Yu Y , Zheng Q , Erramilli SK , Pan M , Park S , Xie Y , Li J , Fei J , Kossiakoff AA , Liu L *et al*. (2021) K29‐linked ubiquitin signaling regulates proteotoxic stress response and cell cycle. Nat Chem Biol 17, 896–905. 34239127 10.1038/s41589-021-00823-5 PMC8717942

[13] Finley D (2009) Recognition and processing of ubiquitin‐protein conjugates by the proteasome. Annu Rev Biochem 78, 477–513. 19489727 10.1146/annurev.biochem.78.081507.101607 PMC3431160

[14] Collins GA and Goldberg AL (2017) The logic of the 26S proteasome. Cell 169, 792–806. 28525752 10.1016/j.cell.2017.04.023 PMC5609836

[15] Dupont N , Leroy C , Hamaï A and Codogno P (2017) Long‐lived protein degradation during autophagy. Methods Enzymol 588, 31–40. 28237108 10.1016/bs.mie.2016.09.074

[16] Lamark T and Johansen T (2012) Aggrephagy: selective disposal of protein aggregates by macroautophagy. Int J Cell Biol 2012, 736905. 22518139 10.1155/2012/736905 PMC3320095

[17] Wang J , Zhang Q , Bao Y and Bassham DC (2023) Autophagic degradation of membrane‐bound organelles in plants. Biosci Rep 43, BSR20221204. 36562332 10.1042/BSR20221204 PMC9842949

[18] Fleming A , Bourdenx M , Fujimaki M , Karabiyik C , Krause GJ , Lopez A , Martín‐Segura A , Puri C , Scrivo A , Skidmore J *et al*. (2022) The different autophagy degradation pathways and neurodegeneration. Neuron 110, 935–966. 35134347 10.1016/j.neuron.2022.01.017 PMC8930707

[19] Li Y , Li S and Wu H (2022) Ubiquitination‐proteasome system (UPS) and autophagy two Main protein degradation machineries in response to cell stress. Cells 11, 851. 35269473 10.3390/cells11050851 PMC8909305

[20] Guo J , Liu J , Wei Q , Wang R , Yang W , Ma Y , Chen G and Yu Y (2017) Proteomes and ubiquitylomes analysis reveals the involvement of ubiquitination in protein degradation in Petunias. Plant Physiol 173, 668–687. 27810942 10.1104/pp.16.00795 PMC5210702

[21] He D , Li M , Damaris RN , Bu C , Xue J and Yang P (2020) Quantitative ubiquitylomics approach for characterizing the dynamic change and extensive modulation of ubiquitylation in rice seed germination. Plant J 101, 1430–1447. 31677306 10.1111/tpj.14593

[22] Liu H , Zhang J , Li J , Yu B , Chen S , Ma C and Li H (2022) Comparative ubiquitination proteomics revealed the salt tolerance mechanism in sugar beet monomeric additional line M14. Int J Mol Sci 23, 16088. 36555729 10.3390/ijms232416088 PMC9782053

[23] Lu J , Xu Y , Fan Y , Wang Y , Zhang G , Liang Y , Jiang C , Hong B , Gao J and Ma C (2019) Proteome and ubiquitome changes during rose petal senescence. Int J Mol Sci 20, 6108. 31817087 10.3390/ijms20246108 PMC6940906

[24] Abreha MH , Dammer EB , Ping L , Zhang T , Duong DM , Gearing M , Lah JJ , Levey AI and Seyfried NT (2018) Quantitative analysis of the brain Ubiquitylome in Alzheimer's disease. Proteomics 18, e1800108. 30230243 10.1002/pmic.201800108 PMC6283072

[25] Baehr LM , Hughes DC , Lynch SA , Van Haver D , Maia TM , Marshall AG , Radoshevich L , Impens F , Waddell DS and Bodine SC (2021) Identification of the MuRF1 skeletal muscle Ubiquitylome through quantitative proteomics. Function 2, zqab029. 34179788 10.1093/function/zqab029 PMC8218097

[26] Dybas JM , O'Leary CE , Ding H , Spruce LA , Seeholzer SH and Oliver PM (2019) Integrative proteomics reveals an increase in non‐degradative ubiquitylation in activated CD4+ *T* cells. Nat Immunol 20, 747–755. 31061531 10.1038/s41590-019-0381-6 PMC7007700

[27] Iesmantavicius V , Weinert BT and Choudhary C (2014) Convergence of ubiquitylation and phosphorylation signaling in rapamycin‐treated yeast cells. Mol Cell Proteomics 13, 1979–1992. 24961812 10.1074/mcp.O113.035683 PMC4125731

[28] Akimov V , Barrio‐Hernandez I , Hansen SVF , Hallenborg P , Pedersen A‐K , Bekker‐Jensen DB , Puglia M , Christensen SDK , Vanselow JT , Nielsen MM *et al*. (2018) UbiSite approach for comprehensive mapping of lysine and N‐terminal ubiquitination sites. Nat Struct Mol Biol 25, 631–640. 29967540 10.1038/s41594-018-0084-y

[29] Kim W , Bennett EJ , Huttlin EL , Guo A , Li J , Possemato A , Sowa ME , Rad R , Rush J , Comb MJ *et al*. (2011) Systematic and quantitative assessment of the ubiquitin modified proteome. Mol Cell 44, 325–340. 21906983 10.1016/j.molcel.2011.08.025 PMC3200427

[30] Agarwal E , Goldman AR , Tang H‐Y , Kossenkov AV , Ghosh JC , Languino LR , Vaira V , Speicher DW and Altieri DC (2021) A cancer ubiquitome landscape identifies metabolic reprogramming as target of Parkin tumor suppression. Sci Adv 7, eabg7287. 34433563 10.1126/sciadv.abg7287 PMC8386929

[31] Rusilowicz‐Jones EV , Jardine J , Kallinos A , Pinto‐Fernandez A , Guenther F , Giurrandino M , Barone FG , McCarron K , Burke CJ , Murad A *et al*. (2020) USP30 sets a trigger threshold for PINK1–PARKIN amplification of mitochondrial ubiquitylation. Life Sci Alliance 3, e202000768. 32636217 10.26508/lsa.202000768 PMC7362391

[32] Hansen FM , Tanzer MC , Brüning F , Bludau I , Stafford C , Schulman BA , Robles MS , Karayel O and Mann M (2021) Data‐independent acquisition method for ubiquitinome analysis reveals regulation of circadian biology. Nat Commun 12, 254. 33431886 10.1038/s41467-020-20509-1 PMC7801436

[33] Li W , Wang H , Yang Y , Zhao T , Zhang Z , Tian Y , Shi Z , Peng X , Li F , Feng Y *et al*. (2018) Integrative analysis of proteome and Ubiquitylome reveals unique features of lysosomal and endocytic pathways in gefitinib‐resistant non‐small cell lung cancer cells. Proteomics 18, 1700388. 29901268 10.1002/pmic.201700388 PMC6099292

[34] Ordureau A , Paulo JA , Zhang J , An H , Swatek KN , Cannon JR , Wan Q , Komander D and Harper JW (2020) Global landscape and dynamics of Parkin and USP30‐dependent ubiquitylomes in iNeurons during Mitophagic signaling. Mol Cell 77, 1124–1142. 32142685 10.1016/j.molcel.2019.11.013 PMC7098486

[35] Rose CM , Isasa M , Ordureau A , Prado MA , Beausoleil SA , Jedrychowski MP , Finley DJ , Harper JW and Gygi SP (2016) Highly multiplexed quantitative mass spectrometry analysis of ubiquitylomes. Cell Syst 3, 395–403. 27667366 10.1016/j.cels.2016.08.009 PMC5241079

[36] Steger M , Demichev V , Backman M , Ohmayer U , Ihmor P , Müller S , Ralser M and Daub H (2021) Time‐resolved in vivo ubiquitinome profiling by DIA‐MS reveals USP7 targets on a proteome‐wide scale. Nat Commun 12, 5399. 34518535 10.1038/s41467-021-25454-1 PMC8438043

[37] Theurillat J‐PP , Udeshi ND , Errington WJ , Svinkina T , Baca SC , Pop M , Wild PJ , Blattner M , Groner AC , Rubin MA *et al*. (2014) Ubiquitylome analysis identifies dysregulation of effector substrates in SPOP‐mutant prostate cancer. Science 346, 85–89. 25278611 10.1126/science.1250255 PMC4257137

[38] Wu Q , Cheng Z , Zhu J , Xu W , Peng X , Chen C , Li W , Wang F , Cao L , Yi X *et al*. (2015) Suberoylanilide hydroxamic acid treatment reveals Crosstalks among proteome, Ubiquitylome and acetylome in non‐small cell lung cancer A549 cell line. Sci Rep 5, 9520. 25825284 10.1038/srep09520 PMC4379480

[39] Grice GL and Nathan JA (2016) The recognition of ubiquitinated proteins by the proteasome. Cell Mol Life Sci 73, 3497–3506. 27137187 10.1007/s00018-016-2255-5 PMC4980412

[40] Jiang C , Jiang T , Deng S , Yuan C , Liang Y , Li S , Ma C and Gao Y (2022) Integrative analysis of transcriptome, proteome, and ubiquitome changes during rose petal abscission. Front Plant Sci 13, 1041141. 36340335 10.3389/fpls.2022.1041141 PMC9627506

[41] Lan W , Ma W , Zheng S , Qiu Y , Zhang H , Lu H , Zhang Y and Miao Y (2022) Ubiquitome profiling reveals a regulatory pattern of UPL3 with UBP12 on metabolic‐leaf senescence. Life Sci Alliance 5, e202201492. 35926874 10.26508/lsa.202201492 PMC9354775

[42] Liu B , Jiang S , Li M , Xiong X , Zhu M , Li D , Zhao L , Qian L , Zhai L , Li J *et al*. (2018) Proteome‐wide analysis of USP14 substrates revealed its role in hepatosteatosis via stabilization of FASN. Nat Commun 9, 4770. 30425250 10.1038/s41467-018-07185-y PMC6233205

[43] Fournier ML , Paulson A , Pavelka N , Mosley AL , Gaudenz K , Bradford WD , Glynn E , Li H , Sardiu ME , Fleharty B *et al*. (2010) Delayed correlation of mRNA and protein expression in rapamycin‐treated cells and a role for Ggc1 in cellular sensitivity to rapamycin. Mol Cell Proteomics 9, 271–284. 19955083 10.1074/mcp.M900415-MCP200 PMC2830839

[44] Chau V , Tobias JW , Bachmair A , Marriott D , Ecker DJ , Gonda DK and Varshavsky A (1989) A multiubiquitin chain is confined to specific lysine in a targeted short‐lived protein. Science 243, 1576–1583. 2538923 10.1126/science.2538923

[45] Magits W and Sablina AA (2022) The regulation of the protein interaction network by monoubiquitination. Curr Opin Struct Biol 73, 102333. 35176591 10.1016/j.sbi.2022.102333

[46] Xie W , Wang J , Tian S , Zhao H , Cao L , Liang Z , Yang J , Zhao Y , Wang B , Jiang F *et al*. (2024) RNF126‐mediated ubiquitination of FSP1 affects its subcellular localization and ferroptosis. Oncogene 43, 1463–1475. 38514855 10.1038/s41388-024-02949-x

[47] Jacomin A‐C and Dikic I (2024) Membrane remodeling via ubiquitin‐mediated pathways. Cell Chem Biol 31, 1627–1635. 39303699 10.1016/j.chembiol.2024.08.007

[48] Catic A , Collins C , Church GM and Ploegh HL (2004) Preferred in vivo ubiquitination sites. Bioinformatics 20, 3302–3307. 15256413 10.1093/bioinformatics/bth407

[49] Zhang Z , Sie B , Chang A , Leng Y , Nardone C , Timms RT and Elledge SJ (2023) Elucidation of E3 ubiquitin ligase specificity through proteome‐wide internal degron mapping. Mol Cell 83, 3377–3392. 37738965 10.1016/j.molcel.2023.08.022 PMC10594193

[50] Zhang Y , Ai Y , Fan Q , Chen B , Zhang J , Lv Y , Song Y , Zhang H , Guo Z and Xu J (2024) Uncovering the profile of ubiquitination motif in catalytic proteins using sequence contextual features. Anim Zool 1, 131–145.

[51] Mészáros B , Kumar M , Gibson TJ , Uyar B and Dosztányi Z (2017) Degrons in cancer. Sci Signal 10, eaak9982. 28292960 10.1126/scisignal.aak9982

[52] Bachmair A , Finley D and Varshavsky A (1986) In vivo half‐life of a protein is a function of its amino‐terminal residue. Science 234, 179–186. 3018930 10.1126/science.3018930

[53] Elu N , Lectez B , Ramirez J , Osinalde N and Mayor U (2020) Mass spectrometry‐based characterization of Ub‐ and UbL‐modified proteins. In Mass Spectrometry Data Analysis in Proteomics ( Matthiesen R , ed.), pp. 265–276. Springer New York, New York, NY. 10.1007/978-1-4939-9744-2_11 31552633

[54] Miller RM and Smith LM (2023) Overview and considerations in bottom‐up proteomics. Analyst 148, 475–486. 36383138 10.1039/d2an01246d PMC9898146

[55] Deol KK and Strieter ER (2021) The ubiquitin Proteoform problem. Curr Opin Chem Biol 63, 95–104. 33813043 10.1016/j.cbpa.2021.02.015 PMC8384647

[56] Sap KA , Bezstarosti K , Dekkers DHW , Voets O and Demmers JAA (2017) Quantitative proteomics reveals extensive changes in the ubiquitinome after perturbation of the proteasome by targeted dsRNA‐mediated subunit knockdown in drosophila. J Proteome Res 16, 2848–2862. 28665616 10.1021/acs.jproteome.7b00156

[57] Xu Y and Hanson MR (2000) Programmed cell death during pollination‐induced petal senescence in petunia. Plant Physiol 122, 1323–1334. 10759529 10.1104/pp.122.4.1323 PMC58968

[58] Shahri W and Tahir I (2014) Flower senescence: some molecular aspects. Planta 239, 277–297. 24178586 10.1007/s00425-013-1984-z

[59] Poret M , Chandrasekar B , van der Hoorn RAL and Avice J‐C (2016) Characterization of senescence‐associated protease activities involved in the efficient protein remobilization during leaf senescence of winter oilseed rape. Plant Sci 246, 139–153. 26993244 10.1016/j.plantsci.2016.02.011

[60] Roberts I , Murray PF , Passeron S and Barneix AJ (2002) The activity of the 20S proteasome is maintained in detached wheat leaves during senescence in darkness. Plant Physiol Biochem 40, 161–166.

[61] Lin Y‐L , Sung S‐C , Tsai H‐L , Yu T‐T , Radjacommare R , Usharani R , Fatimababy AS , Lin H‐Y , Wang Y‐Y and Fu H (2011) The defective proteasome but not substrate recognition function is responsible for the null phenotypes of the Arabidopsis proteasome subunit RPN10. Plant Cell 23, 2754–2773. 21764993 10.1105/tpc.111.086702 PMC3226219

[62] Lu J , Zhang G , Ma C , Li Y , Jiang C , Wang Y , Zhang B , Wang R , Qiu Y , Ma Y *et al*. (2024) The F‐box protein RhSAF destabilizes the gibberellic acid receptor RhGID1 to mediate ethylene‐induced petal senescence in rose. Plant Cell 36, 1736–1754. 38315889 10.1093/plcell/koae035 PMC11062431

[63] Becker K , Bluhm A , Casas‐Vila N , Dinges N , Dejung M , Sayols S , Kreutz C , Roignant J‐Y , Butter F and Legewie S (2018) Quantifying post‐transcriptional regulation in the development of *Drosophila melanogaster* . Nat Commun 9, 4970. 30478415 10.1038/s41467-018-07455-9 PMC6255845

