# Supplementary figures and images for: Meta‐analysis fails to show any correlation between protein abundance and ubiquitination changes
Source: FEBS Open Bio. 2026 Jan 24;16(6):1074–86. doi: 10.1002/2211-5463.70197 (PMC13238665; doi:10.1002/2211-5463.70197)

Supplementary file 1. PRISMA flow diagram.

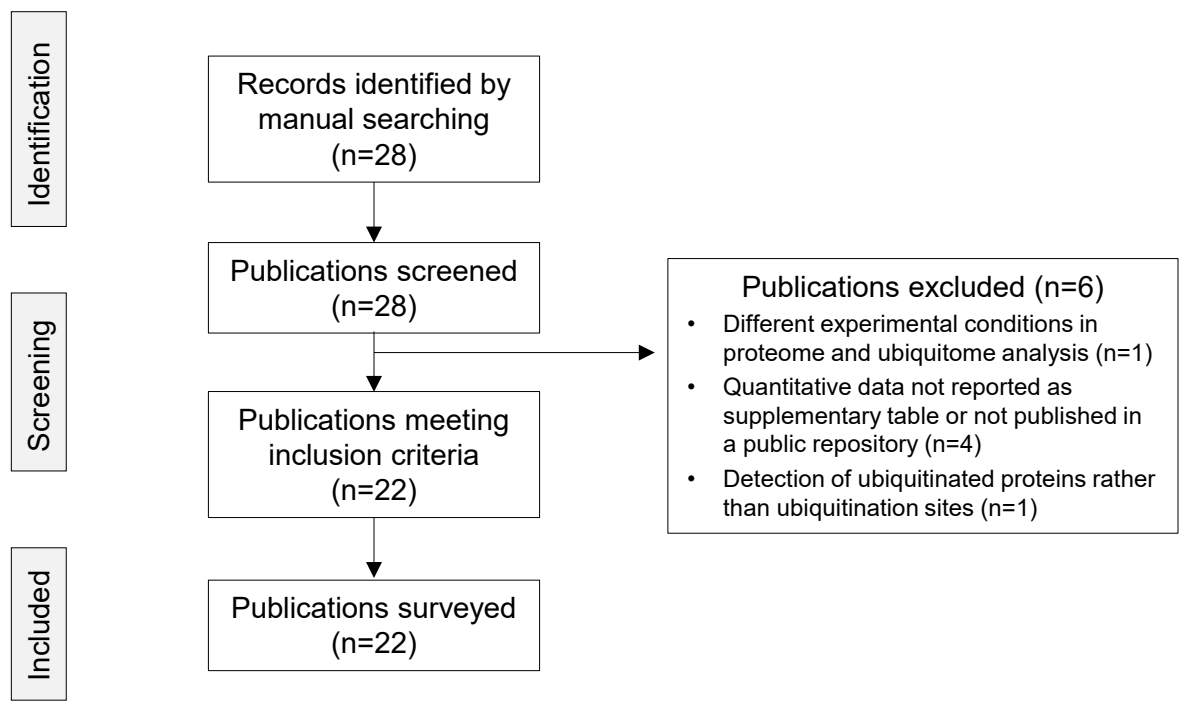

Supplement: Supplementary file 1 — File S1. PRISMA flow diagram. [file FEB4-16-1074-s008.pdf]
