# Supplementary material for: Meta‐analysis fails to show any correlation between protein abundance and ubiquitination changes
Source: FEBS Open Bio. 2026 Jan 24;16(6):1074–86. doi: 10.1002/2211-5463.70197 (PMC13238665; doi:10.1002/2211-5463.70197)
Supplement: Supplementary file 6 — Fig. S1. Protein – diGly correlation analysis for experimental conditions other than the highest‐correlation condition. [file FEB4-16-1074-s004.pdf]

Cell lines

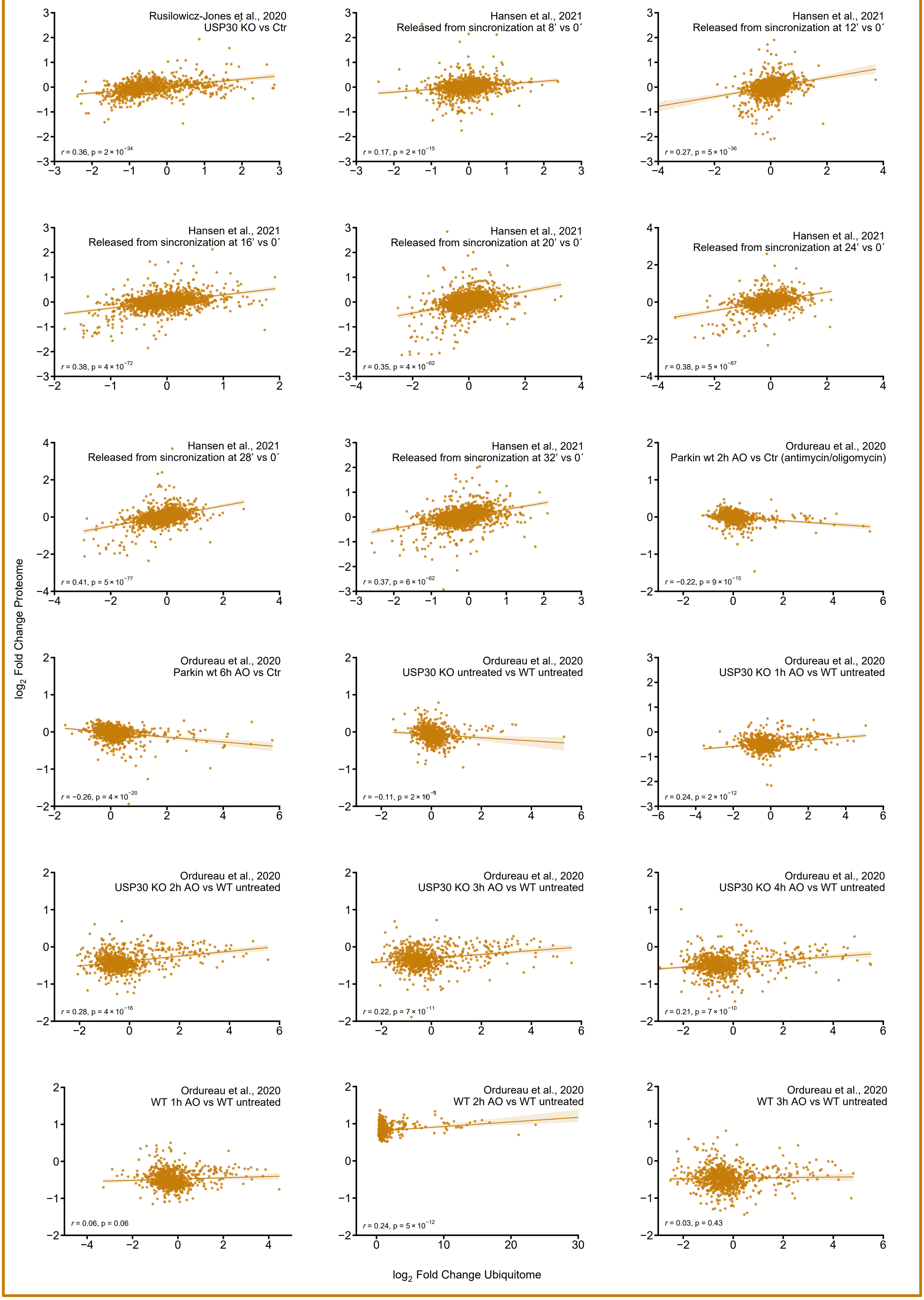

Rusilowicz-Jones et al., 2020
USP30 KO vs Ctr
r = 0.36, p = 2 × 10^−34
Hansen et al., 2021
Released from sincronization at 8’ vs 0´
r = 0.17, p = 2 × 10^−15
Hansen et al., 2021
Released from sincronization at 12’ vs 0´
r = 0.27, p = 5 × 10^−36
Hansen et al., 2021
Released from sincronization at 16’ vs 0´
r = 0.38, p = 4 × 10^−72
Hansen et al., 2021
Released from sincronization at 20’ vs 0´
r = 0.35, p = 4 × 10^−62
Hansen et al., 2021
Released from sincronization at 24’ vs 0´
r = 0.38, p = 5 × 10^−67
Hansen et al., 2021
Released from sincronization at 28’ vs 0´
r = 0.41, p = 5 × 10^−77
Hansen et al., 2021
Released from sincronization at 32’ vs 0´
r = 0.37, p = 6 × 10^−62
Ordureau et al., 2020
Parkin wt 2h AO vs Ctr (antimycin/oligomycin)
r = −0.22, p = 9 × 10^−15
Ordureau et al., 2020
Parkin wt 6h AO vs Ctr
r = −0.26, p = 4 × 10^−20
Ordureau et al., 2020
USP30 KO untreated vs WT untreated
r = −0.11, p = 2 × 10^−3
Ordureau et al., 2020
USP30 KO 1h AO vs WT untreated
r = 0.24, p = 2 × 10^−12
Ordureau et al., 2020
USP30 KO 2h AO vs WT untreated
r = 0.28, p = 4 × 10^−16
Ordureau et al., 2020
USP30 KO 3h AO vs WT untreated
r = 0.22, p = 7 × 10^−11
Ordureau et al., 2020
USP30 KO 4h AO vs WT untreated
r = 0.21, p = 7 × 10^−10
Ordureau et al., 2020
WT 1h AO vs WT untreated
r = 0.06, p = 0.06
Ordureau et al., 2020
WT 2h AO vs WT untreated
r = 0.24, p = 5 × 10^−12
Ordureau et al., 2020
WT 3h AO vs WT untreated
r = 0.03, p = 0.43
log2 Fold Change Proteome
log2 Fold Change Ubiquitome

Cell lines

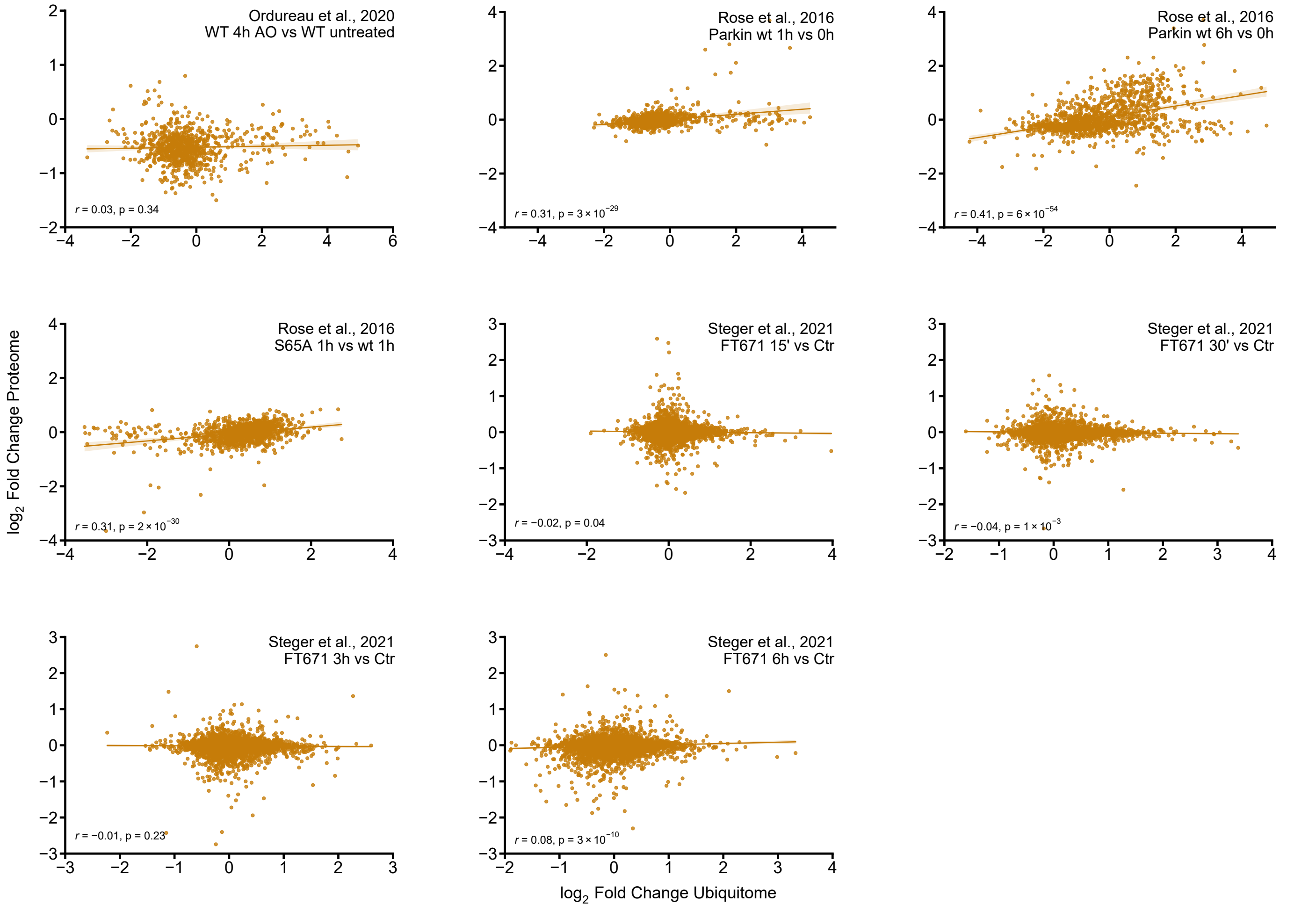
Ordureau et al., 2020
WT 4h AO vs WT untreated
r = 0.03, p = 0.34
Rose et al., 2016
Parkin wt 1h vs 0h
r = 0.31, p = 3 × 10^−29
Rose et al., 2016
Parkin wt 6h vs 0h
r = 0.41, p = 6 × 10^−54
Rose et al., 2016
S65A 1h vs wt 1h
r = 0.31, p = 2 × 10^−30
Steger et al., 2021
FT671 15' vs Ctr
r = −0.02, p = 0.04
Steger et al., 2021
FT671 30' vs Ctr
r = −0.04, p = 1 × 10^−3
Steger et al., 2021
FT671 3h vs Ctr
r = −0.01, p = 0.23
Steger et al., 2021
FT671 6h vs Ctr
r = 0.08, p = 3 × 10^−10
log2 Fold Change Proteome
log2 Fold Change Ubiquitome

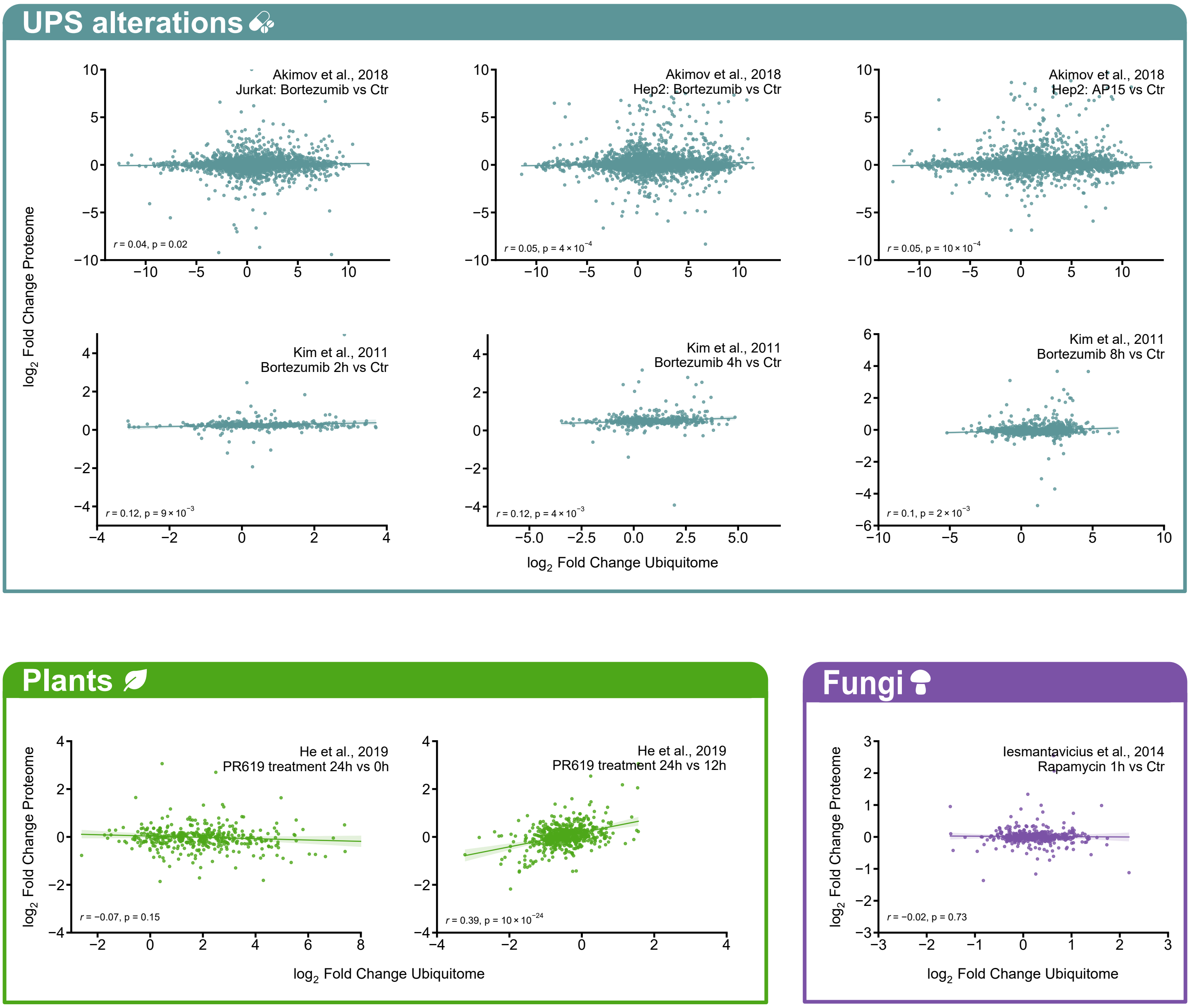

Supplementary Figure 1. In the case of articles with more than one experimental condition, all the conditions but the one displaying the correlation closest to the expected (Figure 1) are shown. For each comparison, Pearson correlation and p-value are calculated.
