# Supplementary material for: Meta‐analysis fails to show any correlation between protein abundance and ubiquitination changes
Source: FEBS Open Bio. 2026 Jan 24;16(6):1074–86. doi: 10.1002/2211-5463.70197 (PMC13238665; doi:10.1002/2211-5463.70197)
Supplement: Supplementary file 7 — Fig. S2. Pearson correlation between proteins and diGly peptides using significantly regulated proteins. [file FEB4-16-1074-s006.pdf]

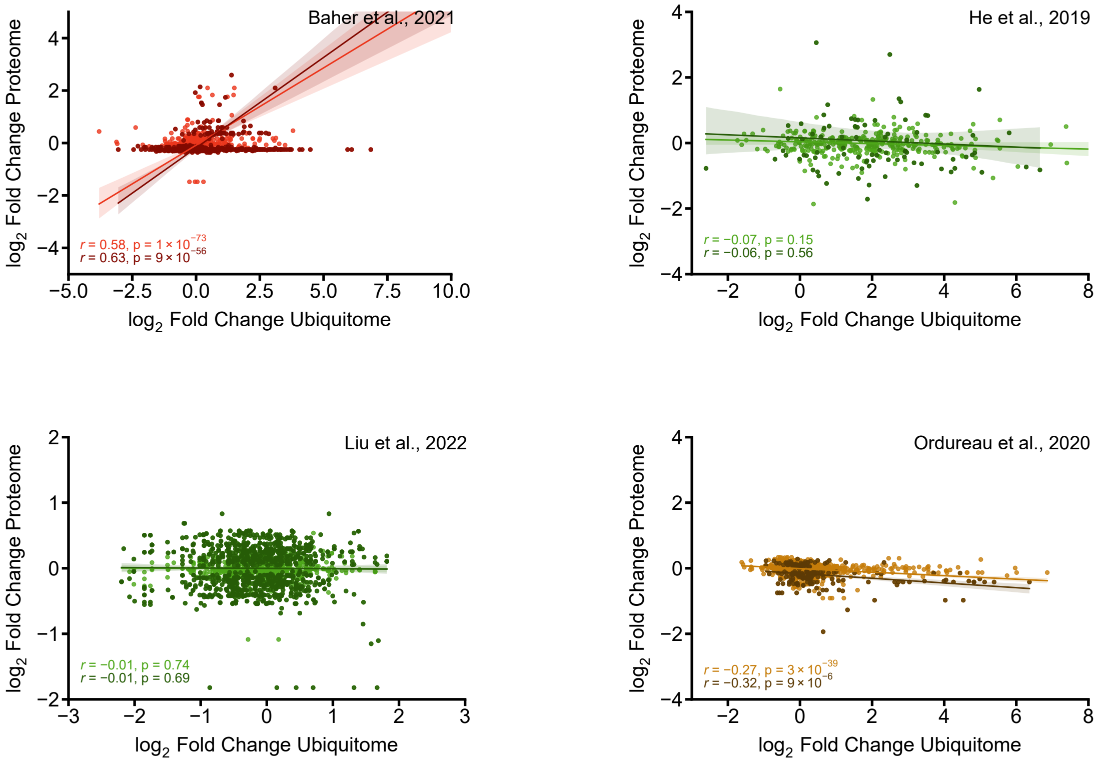

Supplementary Figure 2. Pearson correlation and p-value calculation using data (all data and only data regarding proteins displaying a p-value <0.05) of four additional investigations reporting protein ratio p-value. Confidence interval is shown as shaded area.
