# Supplementary material for: Meta‐analysis fails to show any correlation between protein abundance and ubiquitination changes
Source: FEBS Open Bio. 2026 Jan 24;16(6):1074–86. doi: 10.1002/2211-5463.70197 (PMC13238665; doi:10.1002/2211-5463.70197)
Supplement: Supplementary file 8 — Fig. S3. Relationship between changes in down‐ and up‐regulated proteins and their corresponding diGly ratios. [file FEB4-16-1074-s002.pdf]

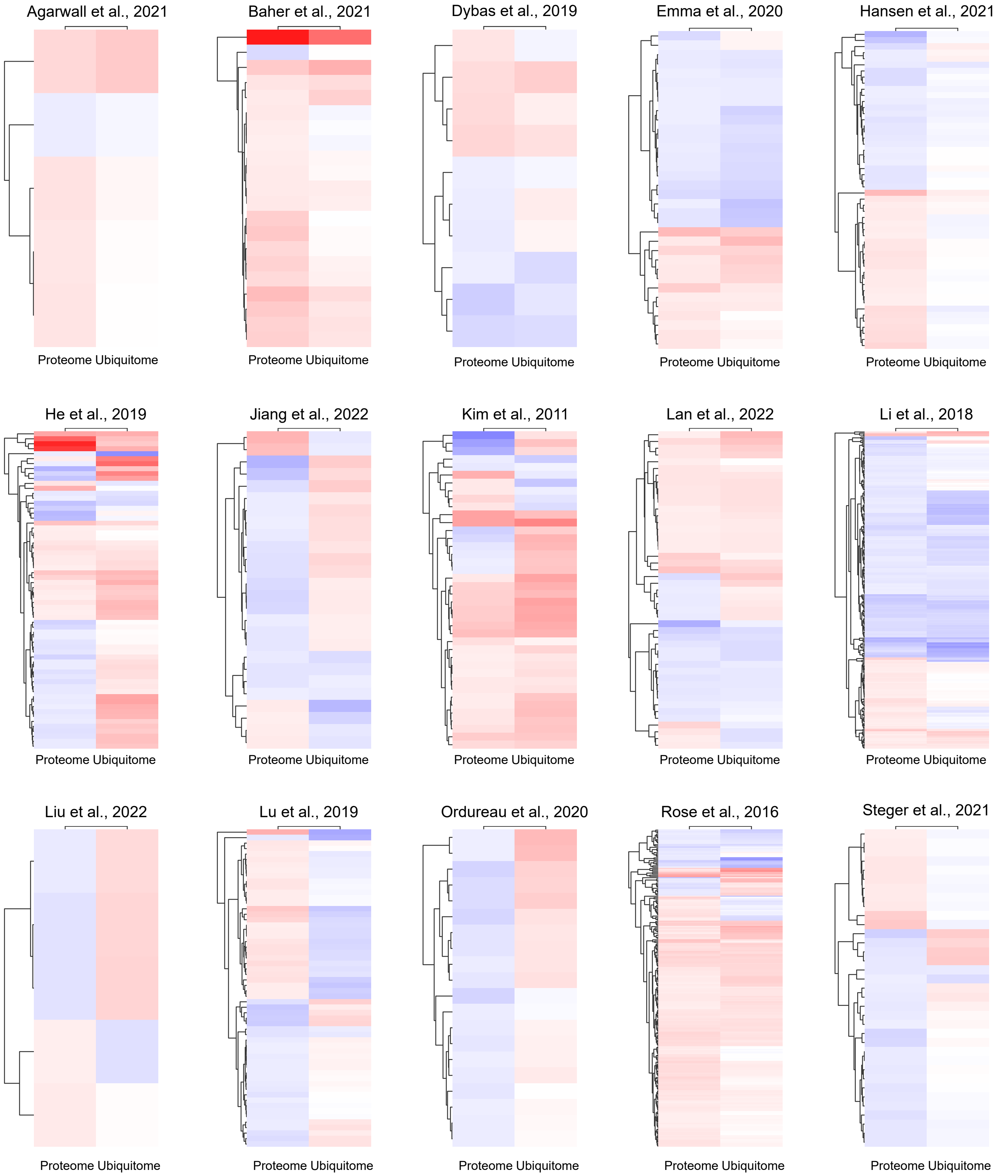

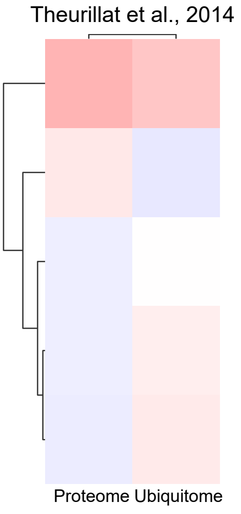

Supplementary Figure 3. Relationship between changes in down- and up-regulated proteins and their corresponding diGly ratios. Hierarchical clustering heat map of proteome and ubiquitome of proteins displaying a fold change above 1.5 or below 0.67. Each row represents a protein from the study. The color code indicates the protein abundance and ubiquitination levels in log2 scale. All experimental conditions analyzed but the representative ones are shown.
